# Wound-Healing Efficacy of *Daucus carota* Bioactive Compounds: Targeting Oxidative Stress, Inflammation, and Apoptosis

**DOI:** 10.3390/ph18121905

**Published:** 2025-12-17

**Authors:** Krishnaraju Venkatesan, Khalid A. Asseri, Pooja Muralidharan, Nizar Sirag, Rehab Ahmed, Hassabelrasoul Elfadil, Mahmoud Elodemi, Shaimaa Elsayed Ramadan Genena, Durgaramani Sivadasan, Malarkodi Velraj, Premalatha Paulsamy, Velmurugan Vadivel, Kousalya Prabahar, Kalpana Krishnaraju

**Affiliations:** 1 Department of Pharmacology, College of Pharmacy, King Khalid University, Abha 62529, Saudi Arabia; kaasseri@kku.edu.sa; 2 Undergraduate Program, PSG College of Pharmacy, Peelamedu, Coimbatore 641004, India; poojaram45232@gmail.com; 3 Department of Natural Products and Alternative Medicine, Faculty of Pharmacy, University of Tabuk, Tabuk 71491, Saudi Arabia; nmona@ut.edu.sa; 4 Division of Microbiology, Immunology and Biotechnology, Department of Natural Products and Alternative Medicine, Faculty of Pharmacy, University of Tabuk, Tabuk 71491, Saudi Arabia; rahmed@ut.edu.sa (R.A.); habdelgadir@ut.edu.sa (H.E.); 5 Pharmacology Department, Faculty of Medicine, University of Tabuk, Tabuk 71491, Saudi Arabia; malodami@ut.edu.sa; 6 Clinical Biochemistry Department, University of Tabuk, Tabuk 71491, Saudi Arabia; sgenena@ut.edu.sa; 7 Department of Pharmaceutics, College of Pharmacy, Jazan University, Jazan 45142, Saudi Arabia; dsivadasa@jazanu.edu.sa; 8 Department of Pharmacognosy, School of Pharmaceutical Sciences, Vels Institute of Science, Technology and Advanced Studies (VISTAS), Chennai 600117, India; malarkodiv.sps@velsuniv.ac.in; 9 Department of Paediatrics, Mahalah Branch for Girls, King Khalid University, Abha 62521, Saudi Arabia; pponnuthai@kku.edu.sa; 10 Department of Pharmaceutical Chemistry, SRM College of Pharmacy, SRM Institute of Science and Technology, Chennai 603203, India; velmuruv@srmist.edu.in; 11 Department of Pharmacy Practice, Faculty of Pharmacy, University of Tabuk, Tabuk 71491, Saudi Arabia; kgopal@ut.edu.sa; 12 Kaarunee VRK Life Sciences, Chennai 600112, India; ezhil@kaaruneevrklifesciences.in

**Keywords:** CSEO, vitamin A, Beta-carotene, anti-inflammatory, antioxidant, inflammatory cytokines

## Abstract

**Background/Objectives**: Carrot seed essential oil (CSEO) from *Daucus carota* is rich in sesquiterpenoids and oxygenated sesquiterpenoids known for anti-inflammatory and antioxidant activities. This study evaluated the wound-healing potential of chemically validated CSEO in albino rats and examined its effects on oxidative stress, inflammation, and apoptosis. **Methods**: CSEO was chemically characterized using GC–MS with retention-index validation. Eighteen constituents (>1% peak area) were identified. An excision wound model in albino rats was used to assess wound closure, physiological parameters, inflammatory cytokines (IL-1β, TNF-α), antioxidant status (SOD, GSH, ROS, MDA), and histological markers of tissue repair. Apoptosis was evaluated through caspase-3 immunohistochemistry to determine its role in tissue remodeling. **Results**: CSEO exhibited an oxygenated sesquiterpene–rich chemotype with oxygenated sesquiterpenes (~36%) and sesquiterpene hydrocarbons (~29%) as major groups. In vivo, CSEO significantly accelerated wound closure, achieving closure on Day 16 (*p* < 0.01) and complete closure by Day 21 (*p* < 0.001), compared with the Reference and Control groups. CSEO significantly improved body weight from Day 4 to Day 21 (*p* < 0.001) and showed consistently higher feed intake (*p* < 0.001) relative to other groups. Inflammatory markers were markedly reduced, with TNF-α and IL-1β significantly lower than in the Control group (*p* < 0.001). CD68 levels were also significantly decreased (*p* < 0.001). CSEO significantly lowered ROS and MDA (*p* < 0.001) while enhancing GSH and SOD levels (*p* < 0.001). Caspase-3 IHC revealed restored physiological apoptotic activity, supporting regulated tissue remodeling. **Conclusions**: CSEO modulates inflammation, oxidative stress, and apoptosis to promote efficient wound healing, supporting its relevance as a promising therapeutic candidate for wound management.

## 1. Introduction

The process of healing wounds is complex and dynamic, involving several biological mechanisms such as tissue remodelling, inflammation, haemostasis, and proliferation. The restoration of skin or other tissues after damage requires a tightly regulated interaction of cellular and molecular components. While the body is typically capable of healing minor wounds on its own, chronic or severe wounds, especially those affected by infection or other underlying conditions, often require therapeutic intervention to promote recovery [1]. Traditional medicine has long utilised various plant-derived essential oils for their wound-healing properties, and one such oil that has recently gained attention is Carrot Seed Essential Oil (CSEO).

CSEO, derived from the seeds of *Daucus carota* L. (Apiaceae), differs chemically from carrot root oil; it is rich not in carotenoids or fatty acids but in volatile sesquiterpenoids such as carotol, daucol, elemol, and bisabolene derivatives. These constituents possess strong antioxidant, anti-inflammatory, and antimicrobial properties, which may collectively contribute to its therapeutic potential in wound healing [2,3]. Carotol, the major oxygenated sesquiterpene, has been reported to exert antifungal, anti-inflammatory, and tissue-regenerative effects through the modulation of cytokines and oxidative pathways [4]. Daucol and elemol exhibit antioxidant and collagen-stimulating activities, while β-bisabolene and γ-cadinene contribute to antioxidant and antimicrobial functions [5]. Together, these bioactive compounds may act synergistically to accelerate tissue repair and protect regenerating cells from oxidative damage.

Earlier reports on CSEO have largely focused on its cosmetic and antimicrobial applications, with limited exploration of its wound-healing mechanisms or chemical-bioactivity correlation [6,7]. The present study is the first to provide a comprehensive, retention-index-validated GC-MS chemoprofile of CSEO, and to link its dominant oxygenated sesquiterpene constituents with experimentally verified wound-healing efficacy in vivo.

Previous studies on carrot extracts and essential oils have reported antioxidant and anti-inflammatory effects that may facilitate wound repair processes such as collagen synthesis and infection control. However, direct evidence for CSEO in wound healing remains limited [8,9].

CSEO appears particularly beneficial in modulating the inflammatory and oxidative phases of wound healing, which are characterised by elevated reactive oxygen species (ROS) and cytokine overproduction. By mitigating oxidative stress and regulating inflammatory signalling, the sesquiterpenoid-rich fraction of CSEO may help maintain the balance required for proper tissue regeneration. Previous studies have demonstrated that essential oils rich in oxygenated sesquiterpenes reduce inflammatory mediators such as TNF-α and IL-1β, enhance antioxidant enzymes (SOD, GSH), and stimulate fibroblast proliferation [10], suggesting similar mechanisms may underpin the bioactivity of CSEO.

The present investigation therefore aimed to characterise the bioactive sesquiterpenoid composition of CSEO using GC-MS coupled with retention-index confirmation and to evaluate its wound-healing effects through biochemical, histological, and molecular markers. Parameters such as wound closure percentage, epithelialisation rate, collagen deposition, and modulation of inflammatory (IL-1β, TNF-α) and oxidative (ROS, MDA, SOD, GSH) indices were used to assess efficacy [11,12,13]. Caspase-3 activity was also monitored to determine anti-apoptotic effects and tissue preservation.

By integrating chemical profiling with in vivo biological validation, this study establishes a novel link between the oxygenated sesquiterpene-rich composition of CSEO and its wound-healing potential. The findings are expected to advance understanding of how specific bioactive sesquiterpenoids contribute to inflammation resolution, oxidative stress reduction, and tissue regeneration, positioning CSEO as a promising natural therapeutic candidate for skin repair [14].

Despite the growing interest in herbal wound-healing agents, there is limited in vivo evidence detailing the mechanistic role of carrot seed essential oil (CSEO) in modulating oxidative stress, inflammatory cytokines, and collagen synthesis during wound repair. This study provides novel insights by integrating biochemical, antioxidant, and histopathological evaluations to establish the therapeutic potential of CSEO in enhancing tissue regeneration.

This study emphasizes CSEO in wound healing. Various parameters, such as measurement of wound contraction, wound epithelization, biochemical estimation, and histopathological evolution, were involved in healing wounds. 

## 2. Result

The findings demonstrate substantial disparities in wound recovery rates across the groups. The CSEO-treated wounds demonstrated a significantly expedited healing process, with enhanced tissue regeneration and faster wound closure compared to the control and reference groups. This highlights that CSEO facilitates a more efficient healing process, reinforcing its potential therapeutic benefits in wound management.

### 2.1. Chemical Characterisation of EO

**Figure 1:**
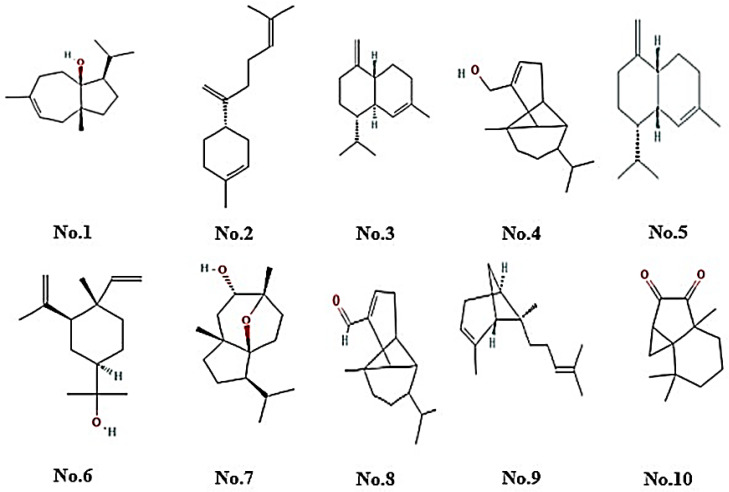
The major phytoconstituents identified in CSEO were as follows—No.1—3A(1H)-Azulenol, 2,3,4,5,8,8a-Hexahydro-6,8a-dimethyl-3-(1-methylethyl)-, No.2—β-Bisabolene, No.3—Naphthalene, 1,2,3,4,4a,5,6,8a-octahydro-7-methyl-4-methylene-1-(1-methylethyl)-(1α,4aβ,8aα), No.4—Tricyclo[4.4.0.0(2,7)]dec-3-ene-3-methanol, 1-methyl-8-(1-methylethyl)-, No.5—(1S,4aR,8aS)-1-Isopropyl-7-methyl-4-methylene-1,2,3,4,4a,5,6,8a-octahydronaphthalene, No.6—Cyclohexanemethanol, 4-ethenyl-α,α,4-trimethyl-3-(1-methylethenyl)-, No.7—Daucol, No.8—3-Ylangenal, No.9—trans-α-Bergamotene, No.10—2H-Cycloprop[c]indene-2,3(3ah)-dione, hexahydro-3a,7,7-trimethyl-.

The CSEO chromatogram revealed 18 compounds (>1% peak area), dominated by oxygenated sesquiterpenes (OS, ≈36%) and sesquiterpene hydrocarbons (SH, ≈29%). Major constituents included carotol (RI 1672, 21.9%), β-bisabolene (RI 1510, 8.5%), and elemol (RI 1686, 2.4%). Minor oxygenated components comprised daucol isomers, ylangenal, and ylangenol (Table 1 and Figure 1). Inclusion of both experimental and reference retention indices confirmed the reliability and reproducibility of compound identification.

### 2.2. Toxicity Studies

The acute cutaneous irritation trial confirmed that CSEO, at a 10% *w*/*w* concentration, was safe, as animals exhibited no visible signs of irritation, redness, erythema, or inflammation throughout the three days of observation.

### 2.3. Measurement of Wound Contraction

**Figure 2:**
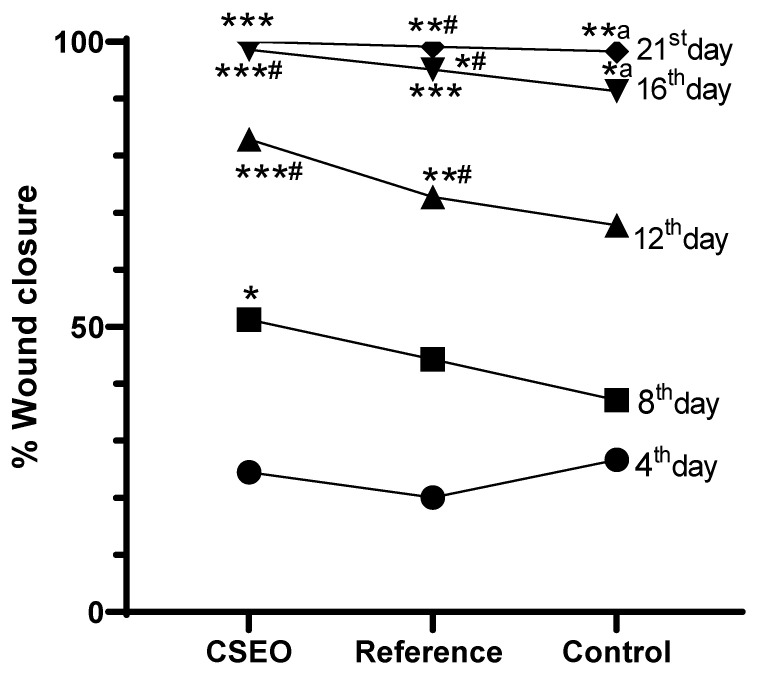
% Wound closure effect on experimental groups. *n* = 6; data are expressed as mean ± SEM. * *p* < 0.01, *^a^ *p* < 0.01, **^a^ *p* < 0.01, ***^#^ *p* < 0.001 indicate statistical significance compared with the control group, *^#^ *p* < 0.01, **^#^ *p* < 0.01, *** *p* < 0.001 indicate statistical significance compared with the reference group. Statistical analysis was performed for all experimental days. Differences among the drug-treated, reference, and control groups on Days 4, 8, and 12 were found to be non-significant (*p* > 0.05) and are therefore not indicated in the figure.

**Figure 3:**
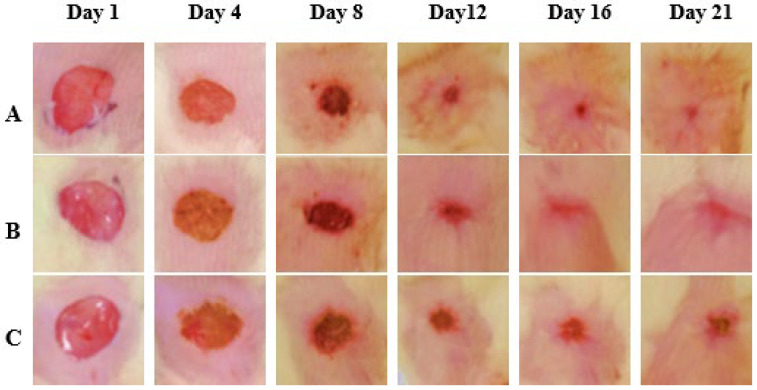
Compared Experimental Group Wound Healing Images. (**A**) CSEO, (**B**) reference, (**C**) Control. Photographs were taken on Days 1, 4, 8, 12, 16, and 21 post-wounding. On Day 1, uniform full-thickness excision wounds were created. By Day 4, wound contraction and scab formation are evident in all groups. The CSEO-treated group (**A**) exhibits enhanced healing, characterised by accelerated wound closure, reduced scab formation, and early tissue regeneration. By Day 21, the CSEO-treated wounds appear nearly healed with minimal scarring, indicating superior efficacy compared to the other two groups—scale bar: 5 mm.

Wound closure % was assessed on the 4th, 8th, 12th, 16th, and 21st days. After treatment, the CSEO-treated group revealed a significant increase in wound closure from the 8th day onwards, reaching near-complete closure by the 21st day. The Reference group additionally exhibited a marked improvement, achieving almost complete closure on the 21st day; however, its effect was modest when compared to the CSEO group. In contrast, the Control group exhibited delayed healing with significantly lower wound closure throughout the study period. These results suggest that CSEO treatment provides superior wound-healing efficacy, surpassing both the Reference and Control groups, ultimately leading to faster and more effective closure (Figure 2 and Figure 3).

### 2.4. Body Weight

**Figure 4:**
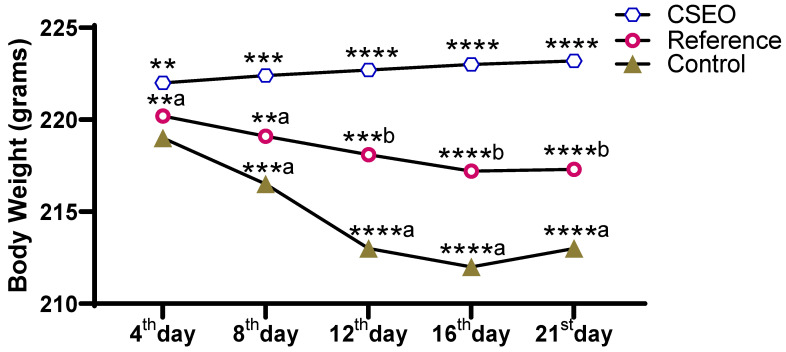
CSEO-Induced Modulation of Body Weight. *n* = 6; data are expressed as mean ± SEM; ** *p* < 0.01, *** *p* < 0.001, **** *p* < 0.0001 indicate statistical significance compared with the control group; **^a^ *p* < 0.01, ***^b^ *p* < 0.001, ****^b^ *p* < 0.0001 indicate statistical significance compared with the Reference group; ***^a^ *p* < 0.001, ****^a^ *p* < 0.0001 indicate statistical significance compared with the reference group.

The body weight changes (Figure 4) were recorded over the experimental period and revealed a clear group-wise variation. The CSEO group demonstrated a consistent increase in body weight from day 4 to day 21, showing highly significant differences at each time point compared with other groups. Conversely, the Reference group had minimal enhancement, with moderate improvements that were statistically inferior to those of CSEO. The Control group showed a progressive decline in body weight across the study period, with the lowest values observed by day 16. These findings indicate that CSEO supplementation effectively maintained and enhanced body weight, whereas the Reference treatment provided only partial protection, and the Control group experienced continuous weight loss.

**Figure 5:**
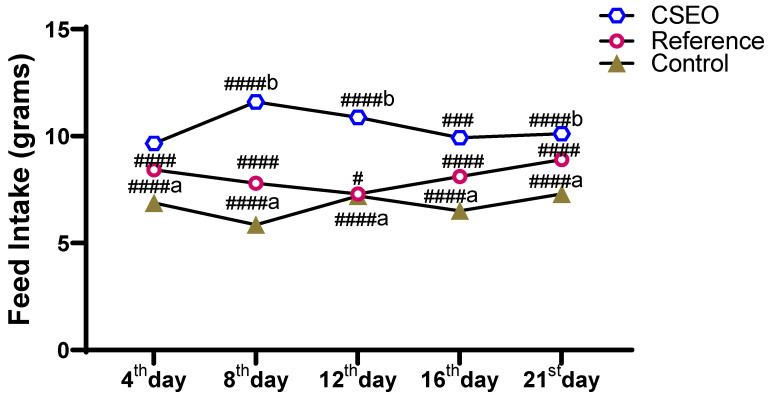
Impact of CSEO on feed intake compared to other groups. *n* = 6; Mean ± SEM, ^####b^ *p* < 0.0001 indicate statistical significance compared with the Control group, ^#^ *p* < 0.01, ^###^ *p* < 0.001, ^####^ *p* < 0.0001 indicate statistical significance compared with the Reference group, ^####a^ *p* < 0.0001—indicate statistical significance compared with the Control group.

Feed intake measurements from Figure 5 demonstrated a distinct difference among the experimental groups throughout the study period. The CSEO group consistently showed the highest feed consumption, with statistically significant increases on all days compared with the reference and control groups. The reference group displayed a moderate rise in intake, remaining significantly lower than the CSEO group but higher than the Control. Conversely, the Control group exhibited the least feed intake, with a noticeable reduction by day 8 and only slight recovery thereafter. These findings confirm that improved feed intake is positively correlated with the body weight gain observed in the CSEO-treated group, which demonstrated superior efficacy in maintaining and improving nutritional status compared to the other two groups.

The variation in body weight observed among the groups was directly influenced by their feed intake. Every day at 8:00 AM, a measured quantity of feed was provided to the animals, and the remaining portion was collected the following morning to determine the daily intake. As illustrated in Figure 5, animals in the CSEO-treated group demonstrated a steady rise in feed consumption, showing statistically significant differences on all experimental days when compared with the other groups. The reference group displayed a slight enhancement, whereas the Control group showed only a minimal increase. These findings indicate a strong association between nutrient intake and gain in body weight, with the CSEO-treated group consistently showing superior performance.

### 2.5. Effect of CSEO on Inflammatory Markers

Table 2 shows that the CSEO-treated group had considerably decreased levels of the inflammatory markers (TNF-α and IL-1β) on day 21 than the reference and control groups. This reduction implies that CSEO successfully regulates the inflammatory response, which promotes better wound healing.

### 2.6. CSEO-Mediated Modulation of CD68 Levels in Experimental Rats

At the end of the study, inflammatory markers (CD68, TNF-α, and IL-1β) were evaluated. In the Control group, CD68 levels were markedly elevated (32.70 ± 1.11 ng/mL), accompanied by high concentrations of TNF-α (660 ± 41.83 pg/mg) and IL-1β (973.3 ± 50.08 pg/mg). Treatment with CSEO significantly reduced CD68 (15.50 ± 1.76 ng/mL), TNF-α (318.3 ± 7.92 pg/mg), and IL-1β (700 ± 28.87 pg/mg), suggesting strong anti-inflammatory potential. The Reference group also showed a decline in CD68 (26.83 ± 0.60 ng/mL), TNF-α (491.7 ± 37.45 pg/mg), and IL-1β (875.0 ± 30.96 pg/mg). However, the magnitude of reduction was modest compared to the CSEO group. These findings clearly indicate that CSEO exerts a superior anti-inflammatory effect by markedly lowering CD68 and TNF-α and IL-1β, thereby outperforming both the Reference and Control groups.

### 2.7. CSEO-Mediated Modulation of Antioxidant Profile

**Figure 6:**
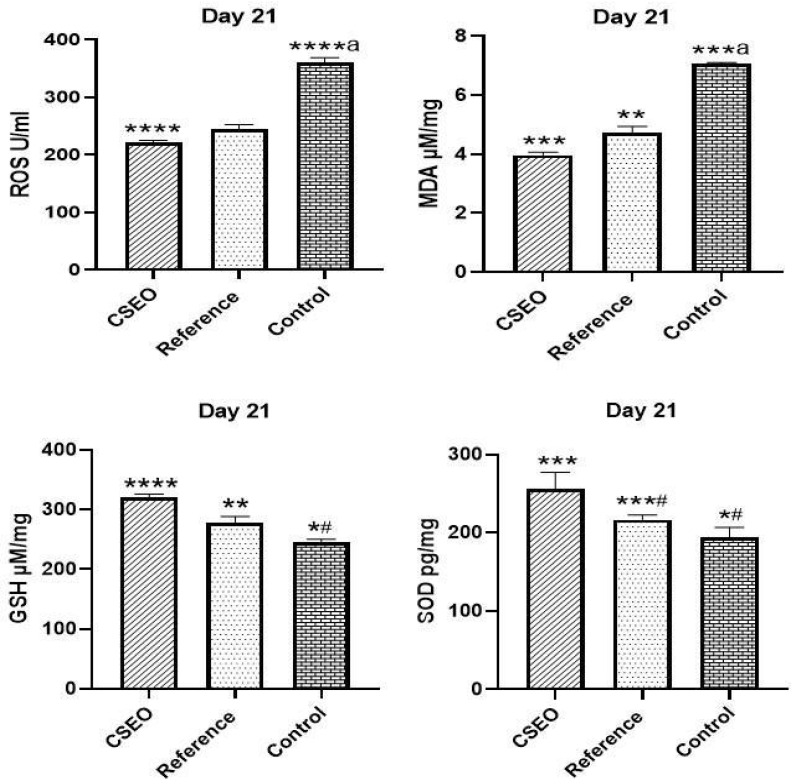
Impact of ROS, GSH, MDA, and SOD among experimental groups. *n* = 6; *** *p* < 0.001, **** *p* < 0.0001 indicate statistical significance compared with the Control group, ** *p* < 0.01, ***^#^ *p* < 0.01, ****^a^ *p* < 0.0001 indicate statistical significance compared with the reference group; *^#^ *p* < 0.01, ***^a^ *p* < 0.001 indicate statistical significance compared with the Control group.

At Day 21, oxidative stress markers were evaluated (Figure 6). In the Control group, a significant elevation in ROS and MDA levels was observed, indicating excessive oxidative burden. After treatment, the CSEO group showed a remarkable reduction in ROS and MDA, suggesting potent antioxidant activity. The Reference group also reduced ROS and MDA levels, but the effect was less pronounced when compared to CSEO. In terms of antioxidant defence, GSH was significantly higher in the CSEO group, demonstrating strong restoration of endogenous antioxidant capacity. The Reference group also increased GSH, while the Control remained significantly lower. Similarly, SOD activity was highest in the CSEO group, followed by the Reference group, whereas the Control showed markedly reduced SOD. Taken together, CSEO treatment effectively reduced ROS and MDA, whereas GSH and SOD were enhanced, proving superior to the reference and Control in restoring redox homeostasis.

### 2.8. Histological Outcome

**Figure 7:**
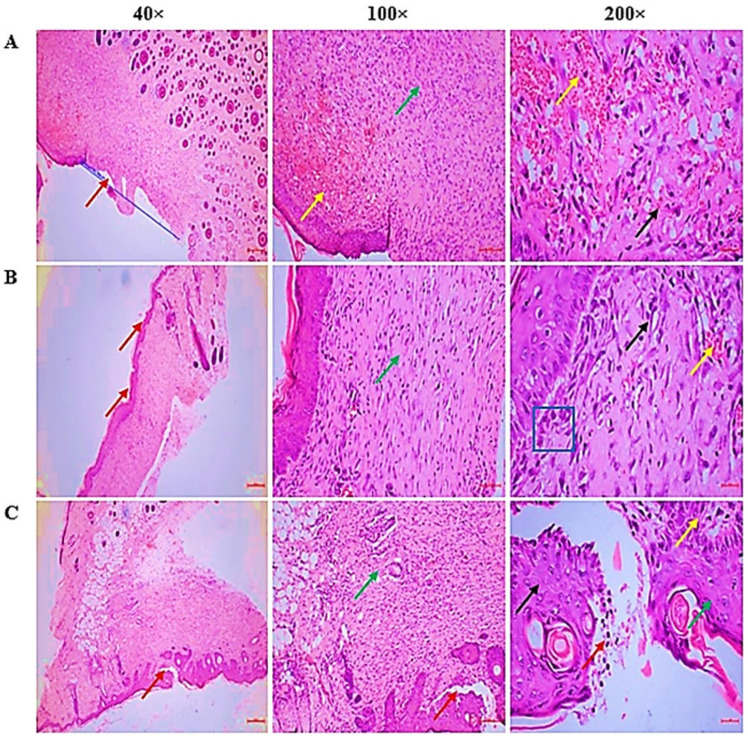
(**A**) CSEO group—The wound area demonstrated complete re-epithelialization (Red arrow). Dermal inflammation was moderate with proliferating granulation tissue (Green arrow) and mild angiogenesis (<1 vessel/HPF) (Yellow arrow) were observed. The granulation tissue contained capillaries (Yellow arrow), producing collagen/ECM, and inflammatory cells (Black arrow), predominantly plasma cells. (**B**) Reference group—complete re-epithelialization with epidermal hyperplasia (Red arrow), moderate dermal inflammation with granulation tissue (Green arrow), and mild angiogenesis (<5 vessels/HPF) (Yellow arrow). The wound bed consisted of capillaries, fibroblasts [Blue square], and inflammatory infiltrates (Black arrow), mainly plasma cells/lymphocytes, with fewer neutrophils. (**C**) Control group—Healing was incomplete, with partial re-epithelialization and accumulation of inflammatory exudates/necrotic debris (Red arrow). A mild inflammatory reaction with granulation tissue (Green arrow) and moderate angiogenesis (5–10 vessels/HPF) (Yellow arrow) was noted. The tissue contained capillaries (Yellow arrow), and inflammatory cells (Black arrow), dominated by neutrophils.

Histopathological examination (Table 3, Figure 7) revealed distinct differences among the groups. CSEO treatment promotes efficient wound repair through improved re-epithelization, granulation, and controlled inflammatory response, while minimising excessive angiogenesis.

### 2.9. IHC

**Figure 8:**
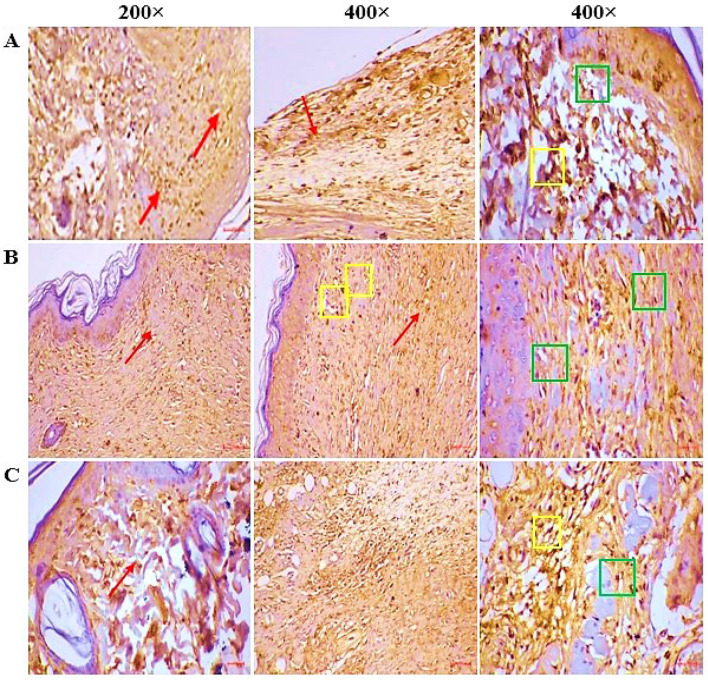
(**A**) CSEO—The fully healed wound site displayed intense caspase-3 activity, predominantly in the epidermal and dermal compartments (red arrow). Strong nucleo-cytoplasmic localization of caspase-3 in the granulation tissue, especially within fibroblasts (green square), as well as in infiltrating immune cells (yellow square). (**B**) Reference—caspase-3 expression was observed at a moderate intensity within both the epidermal and dermal regions (red arrow). The granulation tissue displayed noticeable nucleo-cytoplasmic staining, predominantly localized in fibroblasts (green square) and immune cells, including neutrophils, lymphocytes, and plasma cells (yellow square). (**C**) Control—The regenerated skin exhibited weak caspase-3 activity in the epidermal as well as dermal layers (red arrow). The granulation tissue showed mild nucleo-cytoplasmic positivity, primarily restricted to fibroblasts (green square) and immune cell infiltrates such as neutrophils, lymphocytes, and plasma cells (yellow square).

IHC (Figure 8) shows reduced expression of pro-inflammatory markers in the CSEO-treated group, showing markedly reduced positive staining, indicating suppression of inflammation and enhanced tissue repair compared to reference and control, which exhibit higher marker expression, reflecting persistent inflammatory activity and delayed wound resolution.

## 3. Discussion

Wound healing is a multifaceted process comprising a sequence of coordinated cellular activities designed to restore the integrity of damaged tissues. This process is influenced by several factors, including inflammation, oxidative stress, cytokine production, cellular proliferation, migration, and re-epithelialization. Among the various natural substances that have been explored for their wound-healing properties, CSEO has garnered significant attention due to its potential therapeutic effects [18].

CSEO, as analyzed by GC–MS in this study (Figure 1, Table 1), showed sesquiterpenoids and oxygenated sesquiterpenoids as major constituents, with carotol (isomer A: 5.39%, isomer B: 21.89%), β-bisabolene isomers (4.44% and 8.54%), (E)-β-farnesene (4.07%), and (+)-γ-cadinene (4.04%) being the most abundant. Although carotol remains the dominant compound, our total carotol content of 27.28% is lower than the 66.78% reported by Özcan and Chalchat (2007) in Turkish carrot seed oil [19]. Similarly, our proportion of β-bisabolene (~12.98%) is higher than the ~6.2% reported in wild carrot oil by Aćimović et al. (2016) [15]. These differences likely reflect chemotypic variation driven by geographic origin, seed maturity, and extraction methods. Moreover, Sieniawska et al. (2016) found that carotol content varied widely (19–33%) across commercially sourced oils, which suggests that our sample falls within a broader natural variability [16]. This critical comparison underscores that while our chemical profile aligns in terms of major constituents, its quantitative constitution reveals a distinct chemotype, which may contribute to differential biological activity.

For instance, previous reports have shown carotol content ranging from 30% to 70%, depending on factors such as plant origin, cultivation conditions, and extraction methods [20]. In our study, carotol was detected as the dominant compound, but its proportion differed from some published data. These variations may be attributed to genotypic differences among *Daucus carota* varieties, environmental influences, extraction parameters such as distillation time and temperature, and storage conditions affecting chemical stability [21]. Additionally, the presence of minor compounds such as elemol, daucol, and trans-alpha-bergamotene aligns with previous findings, yet their relative abundance may fluctuate due to differences in soil composition, geographic origin, and post-harvest processing [17]. These chemical constituents have been previously associated with anti-inflammatory, antioxidant, and apoptotic regulatory properties, suggesting their potential role in wound healing. The following sections explore the correlation between CSEO bioactive composition and its mechanistic effects on macrophage activity, inflammatory modulation, oxidative stress regulation, and apoptotic pathways involved in tissue regeneration.

The biological reaction that occurs during wound healing is intricate and involves a planned series of actions, including immune cell activation, inflammation, oxidative stress regulation, cellular defence mechanisms, and tissue remodeling. The GC-MS analysis of CSEO identified key bioactive substances such as carotol, beta-bisabolene, (E)-beta-farnesene, and (+)-gamma-cadinene, which have well-documented anti-inflammatory, antioxidant, and apoptosis-regulating properties. The findings from this study suggest that these bioactive constituents contribute to CSEO’s ability to enhance wound healing by modulating CD68, IL-1β, TNF-α, ROS, MDA, GSH, SOD, and Caspase-3 regulation.

Macrophages are among the first responders in wound healing, playing a critical role in clearing debris, secreting cytokines, and transitioning from M1 (pro-inflammatory) to M2 (anti-inflammatory) phenotypes. CD68, a well-established marker of macrophage activity, was significantly reduced in CSEO-treated wounds, suggesting that CSEO modulates macrophage infiltration and activity, leading to a more controlled immune response. This reduction in CD68 expression aligns with studies showing that terpenoid compounds, particularly carotol and beta-bisabolene, regulate macrophage polarisation, reducing excessive macrophage-driven inflammation and fostering tissue repair [22]. Excessive macrophage infiltration can prolong the inflammatory phase, impair wound resolution, and increase fibrosis [23]. The findings from this study suggest that CSEO supports an optimal immune balance, transitioning the wound microenvironment from a pro-inflammatory to a pro-regenerative state.

As shown in Figure 3, the accelerated wound closure observed in the CSEO-treated group is consistent with its anti-inflammatory and tissue-repair properties. In addition to the untreated control groups, our study included a standard wound-healing reference treatment to enable a more meaningful comparative evaluation of 10% CSEO. The reference formulation widely used as a benchmark in excision-wound studies was applied in the same dosage (0.5 g/day) and served as a positive control for assessing relative efficacy. When compared with this standard treatment, CSEO consistently demonstrated superior wound-healing performance. By Day 21, CSEO achieved complete (100%) wound closure, marginally surpassing the reference group (99.12%). Earlier time point comparisons further highlight this advantage; for example, on Day 12, the CSEO group exhibited 82.9% wound contraction, compared with 72.78% in the reference group. Biochemical parameters showed similar trends, with CSEO producing greater reductions in TNF-α, IL-1β, ROS, and MDA levels, and more pronounced increases in GSH and SOD than the reference treatment. These findings suggest that 10% CSEO not only performs comparably to a recognized standard wound-healing therapy but also provides additional therapeutic benefits, likely due to its rich profile of oxygenated sesquiterpenoids and antioxidant constituents.

Although CSEO was applied topically, the data in Figure 4 and Figure 5 show statistically significant differences in body weight and feed intake between groups. These systemic parameters are not likely a direct pharmacological effect of CSEO; rather, they may reflect secondary improvements associated with reduced wound-related discomfort and stress. Similar observations have been reported in topical wound-healing studies, where improved lesion resolution indirectly enhances feeding behavior and weight stability by alleviating pain and inflammation. In our study, the CSEO-treated mice showed slightly higher mean values than both the reference (Group II) and control groups, but the magnitude of change remained within a biologically acceptable range, suggesting an indirect, supportive effect rather than a systemic therapeutic action. Importantly, these modest systemic differences align with the significantly greater wound-closure percentage observed in the CSEO group compared with the control (Figure 2), reinforcing that the primary contribution of CSEO is local wound repair, with secondary improvements in general well-being.

Inflammatory cytokines, especially TNF-α and IL-1β, are crucial in the inflammatory phase by governing immune cell recruitment and tissue degradation [24]. While a controlled inflammatory response is necessary for pathogen clearance, prolonged IL-1β and TNF-α elevation leads to chronic inflammation, excessive oxidative stress, and delayed healing [25].

In this study, CSEO significantly reduced TNF-α and IL-1β levels, reinforcing its anti-inflammatory properties (Table 2). This modulation of inflammatory mediators may be associated with sesquiterpenoids, which have been reported in previous studies to suppress NF-κB activation, a key driver of cytokine production [26]. Although NF-κB signalling was not directly assessed in this study, such previously documented effects suggest that CSEO may contribute to improved fibroblast migration, enhanced collagen deposition, and accelerated wound closure [27]. Thus, the shift from the inflammatory phase to the proliferative phase observed in CSEO-treated animals is potentially linked to the phytochemical composition of the oil, which could support cellular regeneration and extracellular matrix remodeling.

Oxidative stress significantly affects wound-healing efficacy, as ROS and MDA can impair cellular integrity and hinder fibroblast proliferation [28]. While moderate ROS levels facilitate angiogenesis and fibroblast migration, excessive oxidative stress impairs wound repair by inducing apoptosis and extracellular matrix degradation [29]. In our study, CSEO-treated rats exhibited significantly lower ROS and MDA levels (Figure 6), indicating enhanced redox homeostasis. These effects may be associated with oxygenated sesquiterpenoids such as elemol and trans-α-bergamotene, which have been shown in previous research to upregulate antioxidant enzymes including SOD and GSH [30]. These findings from Figure 3 align with previous research indicating that antioxidant-rich EO protect fibroblasts and keratinocytes from oxidative damage, thereby improving wound repair [29]. Although Nrf2 signalling was not directly evaluated here, earlier studies demonstrating that oxygenated hydrocarbons such as ylangenol and elemol activate the Nrf2 pathway [31,32] imply that CSEO potentially influences similar antioxidant defence mechanisms, thereby improving tissue resilience and reducing oxidative stress–induced apoptosis.

Apoptosis plays a dual role in wound healing. It is essential in the early phase for removing necrotic and damaged cells, but excessive or prolonged activation can impair fibroblast survival and delay epithelialization [33]. Caspase-3, a key executioner of the apoptotic cascade, has been implicated in fibroblast depletion and extracellular matrix degradation [34]. CSEO treatment markedly reduced Caspase-3 activity (Figure 8), suggesting a tendency toward enhanced cell survival and tissue preservation. These effects could be linked to oxygenated sesquiterpenoids present in CSEO, which have been reported to modulate pro-apoptotic proteins such as Bax and anti-apoptotic proteins such as Bcl-2 [35]. However, as apoptotic pathway mediators were not directly measured in this study, these mechanistic explanations should be interpreted as potential rather than confirmed pathways.

Beyond direct interference with the apoptotic machinery, these compounds are also reported to activate cytoprotective and pro-survival pathways, notably the Nrf2–HO-1 antioxidant axis and PI3K/Akt–Bcl-2 signalling [36,37]. Activation of these pathways stabilizes mitochondrial membranes, mitigates oxidative stress, and supports fibroblast proliferation, collagen deposition, and re-epithelialization during the proliferative phase. Thus, the reduction in Caspase-3 activity observed in this study does not imply total inactivation of apoptosis, but rather a phase-specific regulation that favours survival and regenerative signalling, consistent with the physiological transition from the inflammatory to the proliferative stage of wound repair [38]. 

Histopathological analysis (Table 3 and Figure 7) further supported these findings, as CSEO-treated wounds exhibited increased re-epithelialization, improved extracellular matrix integrity, and enhanced neovascularization, suggesting that CSEO facilitates a more structured and efficient wound healing process. This effect can be attributed to CSEO’s ability to reduce inflammation, oxidative stress, and apoptosis while enhancing fibroblast and keratinocyte proliferation.

The findings from this study demonstrate that CSEO exerts significant therapeutic effects on wound healing by modulating immune activity, oxidative stress regulation, apoptosis suppression, and cellular regeneration. The bioactive compounds identified through GC-MS analysis, particularly sesquiterpenoids and oxygenated hydrocarbons, provide mechanistic support for these effects. The results further indicate that CSEO produces clinically meaningful improvements in wound healing, supported by consistent quantitative evidence. By Day 20, CSEO achieved 100% wound closure, surpassing both the reference treatment (99.12%) and the control (98.29%). Accelerated healing was already evident by Day 12, where the CSEO group showed 82.9% closure, compared with 72.78% in the reference and 67.79% in the control groups, demonstrating a faster progression of tissue repair.

Although the 10% *w*/*w* concentration demonstrated clear therapeutic activity, the study did not include multiple concentrations to establish a full dose–response curve. Future work will incorporate graded doses of CSEO (e.g., 2.5%, 5%, 10%, 20%) to determine the minimal effective dose and the optimal therapeutic window.

Biochemical analyses reinforce these therapeutic effects. CSEO significantly reduced oxidative stress, with ROS levels decreasing to 221 U/mL, compared with 244 U/mL in the reference group and 360 U/mL in the control. Lipid peroxidation was similarly attenuated, as evidenced by reduced MDA levels in the CSEO group (3.97 µM/mg) relative to the reference (4.56 µM/mg) and control (7.05 µM/mg). Antioxidant capacity was concurrently enhanced, reflected by elevated GSH (320 µM/mg) and SOD (257 pg/mg) levels in CSEO-treated animals compared with both the reference (GSH 278 µM/mg, SOD 217 pg/mg) and control groups (GSH 246 µM/mg, SOD 194 pg/mg). Moreover, the reduction in Caspase-3 expression suggests decreased apoptosis, contributing to improved tissue integrity and regeneration.

While Caspase-3 inhibition highlights key molecular pathways involved in accelerated healing, further studies are needed to clarify CSEO’s influence on additional regulatory mechanisms, including E-cadherin/N-cadherin dynamics and growth-factor-mediated pathways such as EGFR and PDGFR. These would provide deeper insight into its molecular regulation of tissue repair. Collectively, the improvements observed spanning wound closure, oxidative balance, anti-inflammatory activity, and apoptotic modulation demonstrate that the therapeutic effects of CSEO are sufficiently strong to be considered clinically meaningful, particularly in conditions characterized by chronic inflammation and oxidative stress. Given its diverse bioactive composition, CSEO represents a promising natural therapeutic agent for wound healing. Future research should focus on clinical validation and formulation optimisation to enhance its therapeutic efficacy.

## 4. Methodology

### 4.1. Albino Rats

The primary facility supplied adult male albino rats weighing 150–170 g (King Khalid University, Abha, Saudi Arabia). The temperature was 25 ± 2 °C and the humidity was 55%. Rats were evaluated under 12 h light and dark cycles. Rats were housed individually and provided with unrestricted tap water and standard laboratory chow. All procedures adhered to the guidelines of the Experimental Animal Ethics Committee [39]. The experimental procedure received approval (ECM/2021-5306; Dated 2 May 2021) from the Institutional Animal Ethics Committee (IAEC) of King Khalid University. The rules for the care and use of experimental animals were adhered to during the experiments.

### 4.2. Animal Welfare and Survival Study Considerations

The experiment was conducted over 21 days, after which all animals were humanely euthanised. Each group consisted of six animals, and their well-being was meticulously monitored to ensure ethical treatment. Daily observations were made for any signs of distress, including abnormal behaviour, excessive weight loss (>20%), severe infections, or impaired mobility. In cases of severe suffering, euthanasia was performed following AVMA guidelines, using CO_2_ inhalation as the primary method, with cervical dislocation as a secondary confirmation. Any mortality during the study was thoroughly documented, and necessary steps were taken to investigate and mitigate potential causes, ensuring the highest standards of animal welfare.

### 4.3. The Oil Source

The oil was obtained from the Naturalis Essential Oil Company, located in Manyata tech park, Karnataka, India and was extracted through steam distillation, as specified by the manufacturer. It is 100% natural, free from blends, dilutions, formulations, additives, or technical adjuvants. Manufactured and packaged by TSBT International, Malur, Karnataka, India (Manufacturing Date: August 24, Batch No: BX824).

### 4.4. Identification of Chemicals in CSEO with GC-MS

The composition of chemicals in CSEO was determined using GC-MS using the Shimadzu GC-MS-QP2010 Plus system (Shimadzu Corporation, Kyoto, Japan) and an HP-5MS capillary column (30 m, 0.25 mm i.d., 0.25 μm film thickness, Agilent Technologies, Santa Clara, CA, USA). A 0.22 µm membrane filter was used to filter the material after it had been diluted in hexane (1:10 *v*/*v*) before analysis. A 1.0 µL aliquot was injected in split mode (40:1) at an injector temperature of 250 °C.

Helium served as the carrier gas, flowing at a constant 1.0 mL/min. At 50 °C, the oven temperature program began and remained there for 1 min. The temperature increased by 8 °C every minute until it attained 280 °C, where it was maintained for 2 min. Mass scanning occurred within the range of 40 to 900 *m*/*z*, with the ion source operating at 200 °C and employing electron impact ionisation at 70 eV. The analysis duration was 31 min. The National Institute of Standards and Technology Library, Version 8.0 (Gaithersburg, MD, USA), was used in conjunction with mass spectrum data to ascertain the chemical constituents [39].

Experimental Kovats retention indices (RI_exp_) were calculated using a C_8_–C_30_ n-alkane series under identical chromatographic conditions following the linear temperature-programmed method of van den Dool and Kratz (1963) [40], consistent with current GC–MS protocols [41,42,43,44].

RI_exp_ was determined as:

*RI*_exp_ = 100 · *Z* + 100 · (*t_R_*(*x*) − *t_R_*(*Z*))/(*t_R_*(*Z* + 1) − *t_R_*(*Z*)),

where *t_R_*(*x*) is the analyte retention time and *Z* and *Z* + 1 are the n-alkanes eluting immediately before and after the analyte.

Compound identification was performed by comparing mass spectra with the NIST 17 library, combined with retention-index filtering against literature values for DB-5/HP-5 columns [40,41]. Compounds with Reverse-Match ≥ 800 and |ΔRI| ≤ 20 were reported as putative identifications (Level 2 confidence) [45].

### 4.5. Preparation of Ointments

A 10% *w*/*w* concentration of CSEO was added to yellow soft paraffin purchased from a pharmacy to create the ointments. By preparing the ointments on a slab, levigation was utilised to guarantee a uniformly smooth texture. Yellow soft paraffin served as the negative control, while 1% *w*/*w* SSD ointment served as the standard control [46].

### 4.6. Toxicity Studies

In accordance with OECD Test Guideline 404 [47], ten male Sprague-Dawley rats (250–300 g) were utilized to assess skin irritation. The animals were split up into two groups of five each. Five rats were used as experimental participants, and five more served as controls. With a sterile razor, the rat back hair was cut to around 20 cm in diameter at the lower mid-position. Subsequently, the rats were relocated to alternative homes. The rats endured a continuous day. A 10% Essential Oil (EO) solution was regularly applied to the depilated area of the test individuals. Using non-irritating sticky tape and gauze, the extracts were applied to the skin for an hour. The extracts were then taken out, the skin surface was cleansed with distilled water, and the degree of irritation was assessed. The site was inspected 24 h after the application, and then again 48 and 72 h later. The control rats were administered a topical application of sterile water using sterile cotton that had been adequately moistened with sterile water. The cotton was then enveloped with gauze and hypoallergenic adhesive tape. Erythema and oedema were assessed utilizing the Draize grading system. A score of 1 indicates minimal erythema or oedema, whereas a score of 0 denotes the absence of both. A score of two indicates modest oedema with raised skin at the periphery of the affected area. A score of 4 indicates severe erythema or oedema, whereas a score of 3 signifies moderate to severe erythema or edema [48]. To ensure animal welfare, any animals displaying signs of excessive irritation or distress were removed from the study and given appropriate veterinary care.

### 4.7. Model for Excision Wounds

#### 4.7.1. Experimental Cohorts

To conduct the wound recovery investigation, healthy adult albino rats of 3 groups (*n* = 6 per group) were utilized:

Group I (Treatment)—Received 0.5 g of 10% *w*/*w* CSEO formulation topically.

Group II (Reference)—Received 1% SSD ointment topically.

Group III (Control)—Received yellow soft paraffin topically.

#### 4.7.2. Wounding

Three cohorts of albino rats were assembled for the investigation. Before infliction of injury, the animals had a 12 h fasting period and were sedated with i.p. of a ketamine (50 mg/kg) and xylazine (10 mg/kg) combination. The rats were positioned supine on a surgical table, and the dorsal region between the shoulders was meticulously shaved with an electric clipper. The exposed area was sanitised with 70% ethanol to preserve aseptic conditions. The approach described by Morton and Malone (1972) was used to make excision wounds. A circular full-thickness wound, approximately 2 cm in diameter, was made on the dorsal thoracic region under semi-sterile conditions. The excised tissue included the epidermis, dermis, and hypodermis. Any bleeding was controlled using sterile gauze. Wounds were left uncovered throughout the study without the application of bandages. Rats that showed symptoms of infection or disease were eliminated from the experiment after careful observation of the animals. Each rat was maintained separately to avert cross-contamination. All procedures were performed under sterile conditions, and postoperative analgesics were administered to ensure animal welfare [49].

### 4.8. Therapeutic Intervention

Every day, using a sterile spatula, the test and reference drugs were applied until the epithelium was completely covered. A tampon soaked in physiological serum was used to carefully clean the surfaces of each incision prior to the administration of therapies. On days 1, 4, 8, 12, 16, and 21, the recorded wound areas were quantified using graph paper [50]. On day 21, the healed wound and adjacent skin were excised and subsequently divided into two equal segments for histopathological analysis and assessment of hydroxyproline levels.

Throughout the study, rats were monitored twice daily for signs of infection, excessive pain, or distress. Any animal exhibiting severe wound infection, prolonged healing failure, or extreme distress was humanely euthanised following institutional guidelines.

### 4.9. Evaluation

*Wound contraction* %: The assessment of wound contraction was utilized for evaluation. To calculate the wound closure rate, the wound was traced every four days with transparent paper and a permanent marker (days 1, 4, 8, 12, 16, and 21). The area of each trace (mm^2^) within its boundaries was computed planimetrically. All wounds were digitally documented at consistent intervals. Adobe Photoshop 2023 (version 24.x) was utilised to quantify the wound area [51].

(1) $$wound\;contraction\%=\frac{wound\;area\;on\;day\;0-On\;particular\;day\;wound\;area}{wound\;area\;on\;day\;0}\times 100$$

### 4.10. Evaluation of Biochemical Parameters

#### 4.10.1. Collecting Samples

Rats in each group were used to assess macroscopic wound incision length, document healing progress through image capture, and collect quantitative data on wound healing. Blood samples were collected using the retro-orbital sinus technique under isoflurane anaesthesia to minimise distress. Serum was extracted using EDTA-free tubes and analysed for IL-1β, TNF-α, and CD68 [52]. Blood collection was limited to no more than 7.5–10% of total blood volume per week to ensure animal welfare.

#### 4.10.2. TNF-α and IL-1β Concentrations Assessments in Rat Serum

The quantities of TNF-α and IL-1β in rat serum were quantified with a commercial enzyme-linked immunosorbent assay (ELISA) kit, in accordance with the manufacturer’s instructions. A standard curve was utilized to assess cytokine concentrations, which were thereafter expressed in pg/mL [53].

#### 4.10.3. CD68 Estimation in Serum of Rat

A commercial ELISA kit was used to quantify the CD68 concentration in rat serum, following the manufacturer’s instructions. The quantity of CD68 was expressed in nanograms per millilitre (ng/mL) [54].

#### 4.10.4. Antioxidant Activity

Skin samples were homogenised in 1 mL of saline per gram of tissue using a glass homogeniser. Before analysis, the supernatant was preserved at −80 °C after centrifugation of the homogenates at 10,000 *g* for 30 min at 4 °C. ELISA kits (Andy Hua Tai, Nanjing, China) were employed to quantify the levels of ROS, MDA, GSH, and SOD protein (antioxidant enzymes) in accordance with the manufacturer’s instructions [55].

### 4.11. Evaluation of Histopathology

#### 4.11.1. Collecting Samples

After being extracted from each animal, samples of the dorsal epidermis were preserved in buffered formalin, processed through gradients of alcohol and xylene, and then embedded in paraffin blocks. Collagen fibre density was measured by staining four µm thick tissue sections with hematoxylin/eosin. For slide analysis and imaging, the Leica Application Suite (Leica Microsystems, Wetzlar, Germany) was used [56].

#### 4.11.2. Immunohistochemical Staining (IHC)

Hematoxylin counterstaining, DAB staining, and antibody-based Caspase-3 immunohistochemistry labelling ([EPR18297], Abcam; Santa Cruz Biotechnology, Santa Cruz, CA, USA) were utilized to identify apoptotic cells in wound tissue. Positive cells displayed a brownish-yellow colour. For each animal, three tissue sections were analysed, and the number of positive cells was quantified at five designated areas within the wound tissue. To take the measurements, Image-Pro Plus 6.0 was used. To compute % of caspase-3+ cells, the number of caspase-3+ cells was normalized to the total cell count [57].

### 4.12. Statistical Analysis

To illustrate the data, the mean ± SEM was employed. GraphPad Prism (version 8) was used to conduct a one-way ANOVA on the data, which was then subjected to Tukey’s multiple comparison test. The significance level was established at *p* < 0.01.

## 5. Conclusions

The findings from this study suggest that CSEO exerts multifaceted wound-healing effects through its bioactive oxygenated sesquiterpenoids, which may modulate immune activity, oxidative stress, apoptosis, and tissue regeneration. The GC-MS and retention-index-validated chemical profiling indicate a distinct oxygenated-sesquiterpene–rich chemotype dominated by carotol, daucol, elemol, β-bisabolene, and γ-cadinene, differentiating it from previously reported carrot seed oils. These constituents, individually and synergistically, appear to contribute to anti-inflammatory (via down-regulation of IL-1β and TNF-α), antioxidant (enhanced SOD and GSH activity, reduced ROS and MDA), and anti-apoptotic (suppressed Caspase-3 activation) mechanisms, thereby potentially supporting fibroblast proliferation, collagen deposition, and epithelial regeneration. Notably, although a few essential oils have previously been shown to influence caspase pathways in wound or inflammation models, this study indicates for the first time that a chemically validated, oxygenated-sesquiterpene-rich CSEO promotes wound healing through controlled caspase-3 modulation in vivo. Such compositional and mechanistic integration supports the novelty of this work, as it suggests a direct link between the chemical chemotype of CSEO and its biological efficacy in wound healing, a relationship rarely explored in earlier literature.

Collectively, these results indicate that CSEO is a bioactive, region-specific essential oil with therapeutic potential in managing wounds associated with chronic inflammation and oxidative stress. The study contributes to current understanding by correlating the oxygenated-sesquiterpene-dominant composition of CSEO with in vivo functional outcomes, setting it apart from prior descriptive or non-validated compositional reports. Future research should focus on delineating the molecular signalling pathways involved (e.g., Nrf2/HO-1, NF-κB, and MAPK cascades) and on formulation optimization for clinical translation and controlled-release topical delivery systems.

## Figures and Tables

**Table 1:** Compounds identified from Carrot Seed essential oil (CSEO) using GC–MS (≥1% peak area).

No	Compound (Preferred Name/Isomer)	RT (min)	RI_exp_	Ri_ref_ ^a^	ΔRI	Match/Rev-Match	Molecular Formula	Chemical Class	Peak Area (%)
1	(+)-γ-Cadinene	14.03	1456	1458	−2	875/892	C_15_H_24_	SH	4.04
2	β-Caryophyllene	14.71	1427	1428	−1	883/897	C_15_H_24_	SH	1.39
3	α-Bergamotene (trans-)	14.89	1472	1474	−2	858/874	C_15_H_24_	SH	1.87
4	(–)-D-Germacrene	15.37	1478	1476	+2	874/888	C_15_H_24_	SH	1.36
5	(E)-β-Farnesene	15.18	1482	1484	−2	890/906	C_15_H_24_	SH	4.07
6	β-Bisabolene (isomer A)	16.13	1508	1505	+3	902/915	C_15_H_24_	SH	4.44
7	(S)-β-Bisabolene (isomer B)	16.16	1510	1505	+5	896/910	C_15_H_24_	SH	8.54
8	γ-Amorphene	15.59	1490	1492	−2	861/880	C_15_H_24_	SH	3.46
9	Ylangenol	17.07	1624	1622	+2	873/884	C_15_H_24_O	OS	1.31
10	Carotol (isomer A)	17.68	1660	1656	+4	891/903	C_15_H_26_O	OS	5.39
11	Carotol (isomer B)	18.41	1672	1668	+4	887/903	C_15_H_26_O	OS	21.89
12	Ylangenal	18.48	1679	1678	+1	859/871	C_15_H_22_O	OS (aldehyde)	2.05
13	Daucol (isomer A)	18.62	1680	1680	0	840/865	C_15_H_26_O_2_	OS	1.80
14	Daucol (isomer B)	18.68	1682	1680	+2	845/867	C_15_H_26_O_2_	OS	2.42
15	Elemol	18.94	1686	1689	−3	862/881	C_15_H_26_O	OS	2.42
16	2H-Cycloprop[c]indene-2,3(3aH)-dione derivative	19.08	1743	1745	−2	853/870	C_13_H_18_O_2_	Terpenoid derivative	1.58
17	Bicyclo[3.2.0]heptane-2,6-diol (Z)	20.39	1828	1830	−2	848/864	C_13_H_22_O_3_	Oxygenated terpenoid	1.17
18	Hexahydrofarnesyl acetone	20.93	1833	1838	−5	892/910	C_18_H_36_O	Aliphatic ketone	1.09

RT = retention time (min); RI_exp_ = experimental Kovats retention index calculated using n-alkane standards (C_8_–C_30_) under identical conditions; RI_ref_ = reported retention index on HP-5/DB-5 column from literature ^a^; ΔRI = difference between experimental and reference indices (RI_exp_ − RI_ref_); Match/Rev-Match = mass-spectral similarity indices from NIST 17 library [15,16], and related essential-oil databases. OS = oxygenated sesquiterpene; SH = sesquiterpene hydrocarbon. Identification confidence corresponds to Schymanski Level 2 (MS + RI confirmation) [17].

**Table 2:** Effect of CD68, TNF-α and IL-1β among experimental groups.

Parameters	CSEO	Reference	Control
CD68 (ng/mL)	15.50 ± 1.7638 ^AAA^	26.83 ± 0.6009 ^AAA^*	32.70 ± 1.112 ^AA^
TNF-α (pg/mg)	318.3 ± 7.923 ^AAA^	491.7 ± 37.45 ^AA^*	660 ± 41.83 ^A^
IL-1β (pg/mg)	700 ± 28.87 ^AAA^	875.0 ± 30.96 ^A^*	973.3 ± 50.08

*n* = 6; Significance was denoted as follows: ^AAA^ *p* < 0.001 drug vs. control, ^AAA^* *p* < 0.001 drug vs. reference group, ^AA^ *p* < 0.01 reference vs. control group, ^AA^* *p* < 0.001 drug vs. reference group, ^A^ *p* < 0.001 reference vs. control group, ^A^* *p* < 0.01 drug vs. reference.

**Table 3:** Changes in CSEO histopathological features among experimental groups.

Score Average	Histopathological Feature				
	Re-Epithelization	Angiogenesis	Inflammatory Response	Collagen Deposition	Granulation Tissue
CSEO	3	1	2	1	2
Reference	2	2	1	1	1
Control	1	3	1	1	1

*n* = 6; Histopathological changes for re-epithelization, angiogenesis, granulation tissue, collagen deposition and inflammatory response.

## Data Availability

The original contributions presented in this study are included in the article. Further inquiries can be directed to the corresponding author.
